# Anæsthesia

**Published:** 1927

**Authors:** A. L. Flemming

**Affiliations:** Hon. Anæsthetist, Bristol Royal Infirmary; Lecturer in Anæsthetics, University of Bristol


### The Bristol Medico-Chirurgical Journal

*"Scire est nescire, nisi id me*
*Scire alius sciret."*

SPRING, 1927.

### ANÆSTHESIA.

Presidential Address, delivered on 13th October, 1926, at the opening of the fifty-fourth Session of the Bristol Medico-Chirurgical Society.

BY

A. L. Flemming, M.B., B.Ch.,
*Hon. Anæsthetist, Bristol Royal Infirmary; Lecturer in Anæsthetics, University of Bristol.*

INDEX THROUGH

---

When nominated as President-elect of this Society I naturally felt diffidence in undertaking so responsible a post, especially as at that time the highest traditions of the office were being maintained by Mr. Carwardine with such inimitable ability; but two irresistible forces compelled me to accept the honour so generously proffered; one was the personal gratification inseparable from such promotion, the other was a conviction that by accepting the invitation I should be acknowledging a great compliment paid by you to that particular branch of medical practice in which I happen to be especially interested.

It seems peculiarly opportune that the day of this meeting should almost coincide with the 80th anniversary of that epoch-making occasion which Weir Mitchell[1] once referred to as "the hour when one eager brain, with Godlike will, decreed the death of pain."

On the 30th September, 1846, in his own office in Boston, and a few days later in the Massachusetts hospital, Morton successfully anæsthetised with ether two patients, and, by so doing, had solved, with dramatic suddenness, a problem which had occupied men's minds for thousands of years.

Within three months of this discovery in America, the new method was established in London, and in a short time it had spread from this centre to many of the countries in Europe; indeed, it was inevitable that an invention of this significance should be welcomed with the utmost enthusiasm in view of the terrifying conditions hitherto prevailing in the operating theatre.

We learn from the writings of Dioscorides[2] that, as long ago as the early part of the first century of the Christian era, it was the practice of some surgeons to administer soothing potions to their patients before operation, and we have no reason to think that in former days mankind was devoid of a sense of mercy, for we read that amongst some people, as the Chinese and the Jews, it was customary to give narcotic drugs to criminals before they were executed.

The drugs commonly used in surgery were mandragora, cannabis indica, hellebore, and poppy, and there can be no doubt but that the drowsiness which these produced rendered the patient more amenable to the process of being securely tied to the operating table, or of being held down by stalwart assistants; but there is abundance of evidence to show that this sleep was only too quickly dispelled by the first stroke of the surgeon's knife, and the scenes of struggling and screaming and superhuman bravery which followed were, according to many authorities, so harassing that everybody in the operating room would be overcome with emotion ; even such a vivid description of a major operation upon a conscious patient as occurs in Dr. Brown's classical *Rab and his Friends*[3] when the wife of James Noble was operated on for tumour of the breast can but imperfectly convey to us, who have never witnessed such events, an idea of the conditions which handicapped the surgeon in his work in those pre-anæsthetic days.

The introduction of ether anæsthesia, destined to abolish such scenes as the above for ever, was the outcome of co-operation between scientist and clinician ; it was Jackson, a scientific chemist, who suggested ether to Morton as a drug which he knew to be capable of producing unconsciousness ; but it was for Morton, the dentist, to prove to the world that this state of oblivion could be maintained during surgical procedures which would otherwise be painful ; furthermore, it is no exaggeration to say that every advance that has subsequently taken place in the art of anæsthesia has been based upon the results of experiments and investigations conducted by scientific workers.

Anæsthesia, as a matter of fact, has been especially fortunate in the large number of authorities in various branches of science who have contributed toward her development. And Bristolians may well be proud to remember that it was in Dr. Beddoes' Pneumatic Institute, in Dowry Square, that the very foundation stone of inhalation anæsthesia was laid by[4] Humphry Davy through his research on the properties of nitrous oxide gas. His experiments on animals were so complete and precise that he was able to show exactly what proportion of oxygen must be added to the gas to render its inhalation harmless ; it was by inhaling nitrous oxide, when suffering from an alveolar abscess, and finding that the pain was completely abolished, that Davy was led to make his famous suggestion that this gas might be used in surgical operations.

Davy's suggestion was not adopted by the profession, which is not altogether surprising when the difficulties of obtaining and accurately administering gas in those days is taken into consideration. But the important fact had been definitely established : — " that unconsciousness could be artificially produced, and maintained, without damage to the individual." It is remarkable that after this nitrous oxide seems to have been regarded as a model of what an anæsthetic should be like, and we find ether and chloroform in many instances recommended as possessing properties similar to those of this agent ; indeed, it was as a substitute for nitrous oxide that ether was recommended to Morton on the memorable occasion in 1846. And it was as a substitute for $N_2O$ that ether was inhaled at those social functions which came to be known as " ether frolics."

I am tempted to quote here two episodes in the story of anæsthesia, which had no effect on its evolution, but which illustrate how easily even sound inventions may fail to reach the public. First, there is the story of Hickman,[5] a surgeon at Ludlow, who in 1822 conducted experiments on animals with carbon dioxide and with nitrous oxide gas, and as a result advocated that anæsthesia should be employed for surgical operations ; but in spite of his appeal to the profession, both in England and in France, his proposals met with nothing but scorn and derision, owing, in all probability, to his judges being ignorant of the value of research work ; or due, perhaps, to the popularity of mesmerism at that time.

Secondly, there is the story of Crawford Long, a practitioner in Jefferson, who had used ether anæsthesia in his own practice during the four years preceding 1846, but had failed to publish his results. This reticence may have been due to a reluctance on his part to draw conclusions from his few cases, only eight in four years ; and he was obviously not a man likely to jump to hasty conclusions, for on one occasion, after amputating a boy's finger under ether, he allowed him to recover consciousness and then amputated another finger to make sure that there was a real difference in the boy's two experiences. He cannot have been lacking in initiative, for he advocated open air and special feeding as a treatment for phthisis.

Moreover, Long was a very busy man with, probably, little opportunity for attending meetings or publishing records of his cases; and that he operated, not as a specialist, but simply as occasion occurred in the course of his general practice, we may conclude from the modest nature of his fees, as evidenced by an account which is still in existence :—[6]

James Venable
to Dr. C. W. Long, Dr.

| 1842. | | | $ |
|---|---|---|---|
| Jan. | 28. | Sulphuric Ether .. .. .. | ·25 |
| Mar. | 30. | Sulph : Ether and Exsecting tumour .. .. .. .. .. | 2·00 |
| May | 13. | Sulph : Ether .. .. .. | ·25 |
| June | 6. | Exsecting tumour .. .. .. | 2·00 |
| | | | $4·50 |

The supremacy of ether which had in a few short months become absolute, was soon to be challenged by chloroform ; not being altogether satisfied with ether because of its disagreeable smell, and difficulty of administration, Simpson searched for a more suitable drug, and once again chemistry came to the assistance of medicine, for Waldie, a chemist of Liverpool, advised Simpson to try pure, chloroform, which he knew had the power of producing sleep.

The incident which convinced Simpson of the potency of this agent must have been a surprise for those who witnessed it. The occurrence took place one evening when Simpson had invited two colleagues to dinner, and finding on his desk a bottle of chloroform which he had sent for, in compliance with Waldie's suggestion, he persuaded his guests to join with him in trying the effect of inhaling some of the drug, each of the three being provided with a little of the precious fluid in a cup ; the result must have been alarming for the rest of the company, for we learn that when the meal was served these three men were found literally, and already, under the mahogany !

Chloroform soon grew in favour in all quarters, except America, and, as was inevitable, it soon began to exact its toll in the form of unexpected deaths.

Possibly these mysterious deaths were the decoy which attracted the interest of physiologists in the first place ; this interest has proved to be the mainstay of the speciality, and it would be a disaster if it should for any reason grow weary or flag. It is, I think, no exaggeration to say that the goal which every modern anæsthetist tries to keep in view is to conduct his administration on purely physiological lines ; in other words, he tries to keep as near to physiology, and as far from pathology, as possible ; his ideal is to keep and to leave the patient normal or physiological; and it is chiefly by observing certain rather complicated reflex phenomena that he gets near his goal.

I think it will be agreed that a profitable field for further research lies in this direction; and that a study of the intrathoracic and intra-abdominal reflexes may lead to a better understanding of such post-operative sequelæ as massive collapse of the lung, and intestinal stasis. The fact that the vagus innervates the stomach and the muscles of the bronchial tubes may have a bearing, and the nerve supply to the musculature of the intestinal canal and its sphincters provides food for thought in connection with stasis following operative manipulation; and in the knowledge that these intestinal symptoms may yield immediately to intrathecal injection of novocain, we have a proof of their having a reflex origin.[7]

Fortunately, at the time of the introduction of ether anæsthesia, there was a practitioner in London to whose scientific mind this subject made especial appeal. Dr. Snow had already gained considerable experimental knowledge of respiration and asphyxia, and was, therefore, well prepared for this new study; and the proof of how successfully he carried out his task lies in the fact that at his death, which occurred eleven years later at the age of forty-five, he left behind him a classical work on anæsthesia remarkable for its fund of practical and theoretical information, based on the deductions which the author had drawn from his own clinical and laboratory observations, practically all of which stand to-day as correct. This was the man who first placed anæsthesia on a scientific footing. He was clearly a man of originality and versatility, capable of grasping whole problems without losing sight of essential detail, one who reminds us of the saying that "men with great thoughts in their heads can think of little things which little men cover with their outspread feet." At one time Snow was deeply interested in the study of epidemics, and was convinced that the infection of cholera was not conveyed by foul smells but existed as a definite poison in water, and was contracted by drinking. On one occasion, in 1854, when cholera had been responsible for 500 deaths in ten days in the parish of St. James, Snow went to the vestry meeting and told the assembly that the prescription for curing the epidemic consisted in removing the handle from the pump in Broad Street; his suggestion met with scorn and derision at the moment, but eventually it was adopted, with the result that the epidemic ceased.

His researches [8] on inhalation led him to investigate the effect of many medicinal remedies administered by this method at the Brompton Hospital; but his most important inquiries were devoted to the subject of inhalation narcosis and were influenced, above all things, as Dr. Richardson [9] remarks in his memoir, by a desire to find "a narcotic vapour which, having the physical properties and practicability of chloroform, should, in its physiological effects, resemble ether in not producing, by any accident of administration, paralysis of the heart."

He fully appreciated the greater safety of ether as compared with chloroform, and being convinced that the reason for the disfavour into which the former drug was falling lay in the want of some special apparatus for its administration, he invented an inhaler which did a good deal to stem the tide of unpopularity.

His investigations into the cause of death from chloroform led him to make experiments to ascertain what percentage of the vapour was necessary to paralyse the heart and the respiration respectively, and this is the piece of work upon which has been built up the art of safe anæsthetising. It is interesting to note that his conclusions were, in the main, confirmed by the committee of the Royal Medical and Chirurgical Society in 1864, also by the British Medical Society Committee in Glasgow, in 1880.

By the time of Snow's death, in 1858, anæsthesia was able to cope with the demands made upon her; but her real struggle to keep pace with surgery was to begin some thirty years later, when the victory over sepsis placed the innermost recesses of the human body at the surgeon's mercy: as a result of this advance, there has arisen an ever-increasing demand upon the power of anæsthesia to assist in the prevention of shock, and in the avoidance of unfavourable sequelæ.

On the whole, I think it must be allowed that the combined efforts of surgeons and anæsthetists have met with a reasonable degree of success in the matter of preventing shock at the time of operation; it must, nowadays, be a rare event for an operation to be abandoned or even unduly hurried, owing to the development of this menace; a matter for congratulation in view of the ever-increasing severity of operative procedure.

It seems to me that the criterion of a good or bad anæsthesia has, in a great measure, been removed from the time of operation to the time *immediately* following it, and that our chief anxiety nowadays must be the avoidance of complications, such as shock, acidosis, and pulmonary affections. So far, our progress in this direction has been uncertain, and we find ourselves still faced by a somewhat dense forest of difficulties; but eventually we shall, no doubt, find our way through with the assistance of our colleagues in other departments of medicine.

If there is one topic more than another in which our different colleagues have shown themselves anxious to come to our assistance, surely it is in the attempt to unravel the confused tangle of conflicting evidence surrounding that mysterious phenomenon, shock.

This is a complication whose origin it may be quite impossible to determine ; it may be caused by surgery, or by anæsthesia, or by both combined. When caused by manipulation, the remedy is naturally not within the sphere of the anæsthetist, but is literally in the hands of the surgeon. When due to anæsthesia, on the other hand, it is due to bad anæsthesia, and the onus is with the administrator. He has either poisoned or asphyxiated his patient, or he has allowed to pass nocuous impulses which he ought to have waylaid *en route.*

The toxic effect of anæsthetics upon the tissues is a recognised fact, and Storm van Leeuwen[10] has shown that impurities in the drugs may greatly increase this risk, but that, on the other hand, even with absolutely pure agents, the tendency is present ; so that there is good reason to regard limitation of dosage as a really important factor in careful administration.

Crile[11] has taken an extreme view in this matter, seeing that he has based his famous anoci-association technique upon the assumption that general anæsthetics, with the exception of nitrous oxide, have no power to stop the flow of centripetal nerve impulses to the brain, and that this can only be affected by blocking the individual nerves concerned.

It is difficult to reconcile this statement with the ordinary events of anæsthesia, with the progressive abolition of reflexes in regular order, according to the depth of narcosis; the question was investigated experimentally by Mann[12] of the Mayo Clinic, and he failed to produce shock in deeply narcotised animals by even prolonged and severe trauma of the limbs.

Perhaps the most common source of difficulty in this direction is met with when certain deep abdominal reflexes call for a more profound degree of narcosis than can be maintained for any length of time without running the risk of overdosing. It is in such instances as this that the combination of local analgesia with general anæsthesia proves so valuable.

This combined method represents the fruit of extensive clinical and laboratory research carried out by two antagonistic schools, the advocates of local and the champions of general anæsthesia; it has the great advantage of being easily adapted to practically all of the many conditions prevailing in surgery; and has advanced the fight against shock to a very appreciable extent. But there is need for still further physiological investigation, especially into the reflexes associated with certain of the branches of the vegetative nervous system, in order that there may be a definite understanding as to how far these nerves can be blocked without risk; experiments, perhaps similar to those of Camus[13] in 1912, by which he was able to show that the respiratory centre could be paralysed with novocaine without the mental processes being affected.

Thanks to the bio-chemist, we have learnt the important lesson that we must regard the state of anæsthesia as one in which there is a lowered blood alkalinity.

The tendency of chloroform anæsthesia to be followed by acidosis was the subject of a memorable paper published by Guthrie in 1894, but the full significance of the subject was only appreciated by degrees, as the profession came to realise that the condition could be produced not only by other anæsthetics, but by a number of other, and quite different causes, one of which, it should be remembered, was the systematic starvation of patients to be operated on.

The advance made in our knowledge of this subject is a striking example of the help the anæsthetist has received from physicians and bio-chemists; it has enabled him to lessen materially the risks of operation; and his hand has been still further strengthened of late by the introduction of insulin.

The anticipation of shock, and the evaluation of the patient's powers of resistance, by means of one of the modern methods of interpreting blood pressure readings constitutes another lesson which has resulted from physiologico-surgical investigations. The method used by Moot[14] is a simple form.

The next remarks I have to make are from notes extracted from *The Index Expurgatorius* of the surgeon and anæsthetist; in other words, from records of post-operative pulmonary complications.

No one will deny that these have proved a tough bone of contention, and, shall I say it, of mutual recrimination between surgeons and anæsthetists, but there are signs now of a desire, on the part of the disputants, to bury this bone in neutral soil, and with scientific ceremonial.

The increase in the incidence of bronchitis, pneumonia, and pulmonary embolism in the subjects of high abdominal operations has proved rather difficult to explain, there being so many factors from which to select the culprit. That the anæsthetic, whatever its nature, is not the sole offender, has been confirmed by the experience of surgeons who have rung the changes between local and inhalation methods, without succeeding in completely eliminating the trouble; the truth being, probably, as Finsterer[15] puts it, that neither ether nor chloroform causes pneumonia in itself, but that when given in excess, these drugs have the power to reduce the patient's resistance to infection, as proved by the experiments of Snell. The septic condition present in the lungs of many of the elderly patients operated on nowadays makes this question a very urgent one. Dr. Anderson, Pathologist to the Victoria Infirmary, Glasgow, in 1,831 consecutive post-mortems from the surgical wards, found that no less than 70 per cent. showed signs of chronic lung trouble. When added to this pre-existing focus of sepsis there happens to be a catarrhal epidemic in sway, the chance of infection must be considerable, even in the absence of surgical shock.

Michon and Pollosson[16] held the theory that lung complications are due, much more often than is generally supposed, to small septic emboli, the result of trauma of the mesentery or viscus; in order to avoid damaging the tissues during operation, they refrained from the use of forceps, swabs, or packs as far as possible; moreover, they often operated without gloves so as to preserve the delicacy of touch; and they found that the cases so treated were particularly immune from lung complications.

If eventually we see these dangers eradicated, it will be one of the finest results yet achieved by clinical research.

The evolution of anæsthesia has depended so largely upon physiological experiment and research, that I believe the many problems still awaiting solution would more than occupy the whole time and thought of a surgery-physiologist if such an individual could be created.

### REFERENCES.

[1] Weir Mitchell, *Brit. M. J.*, 1909, ii. 1,084.
[2] Dioscorides, *De Med. Mat.*, bk. iv., § 76.
[3] Brown, *Rab and his Friends* (World's Classics Ed.), p. 273.
[4] Davy, Works of, London, 1839, v. 3, 196–266.
[5] Hickman, *Brit. M. J.*, 1912, i. 843.
[6] Gwathmey's *Anæsthesia*, ed. 2, p. 9.
[7] Asteriades, T., *Presse méd.*, Par. 1925, xxxiii. 1480.
[8] Snow, *Lond. J. M.*, 1851, iii. 122.
[9] Snow, *On Anæsthetics*, Lond., 8vo, 1858, xxviii.
[10] Leeuwen, *Proc. Roy. Soc. Med.*, Lond., Sect. Anæsth., vol. 17, p. 17.
[11] Crile and Lowes, Anoci Association, 1914.
[12] Mann, *Amer. Year Book of Anæsth.*, 1915, 67.
[13] Soupault, *Presse méd.*, Par. 1923, xxxi. 379.
[14] Moot, *Brit. J. Anæsth.*, Manchester, 1926–27, iv. 51.
[15] Finsterer, *Brit. J. Anæsth.*, Manchester, 1924–5, ii. 164.
[16] Delore, Michon et Pollosson, *Presse méd.*, Par. 1924, xxxiii. 762.

